# Tryptophan hydroxylase (TRH) loss of function mutations induce growth and behavioral defects in *Daphnia magna*

**DOI:** 10.1038/s41598-018-19778-0

**Published:** 2018-01-24

**Authors:** Claudia Rivetti, Bruno Campos, Benjamín Piña, Demetrio Raldúa, Yasuhiko Kato, Hajime Watanabe, Carlos Barata

**Affiliations:** 1 Department of Environmental Chemistry, Institute of Environmental Assessment and Water Research (IDAEA, CSIC), Jordi Girona 18, 08017 Barcelona, Spain; 2 Graduate School of Engineering, Osaka University, Suita, Osaka, Japan

## Abstract

Tryptophan hydroxylase (TRH) is the rate limiting enzyme in the serotonin synthesis. CRISPR-Cas9 technology was used to generate seven indel TRH mutants in *Daphnia magna*. Mono-allelic indel TRH−/+ clones showed normal levels of serotonin, measured by both immunohistochemistry and mass spectrometry (LC-MS/MS), whereas bi-allelic indel TRH−/− clones showed no detectable levels of serotonin. Life history and behavioral responses of TRH−/− clones showed the anti-phenotype of those exposed to selective serotonin reuptake inhibitors (SSRI). Mutants lacking serotonin grew less and hence reproduced latter, produced smaller clutches of smaller offspring and responded to a greater extent to light than wild type individuals. Mono-allelic indel TRH−/+ individuals showed the intermediate phenotype. The SSRI fluoxetine enhanced offspring production in all clones and decreased the response to light only in those clones having serotonin, thus indication that behavioral effects of this drug in *D. magna* are associated to serotonin. Results obtained with the TRH mutants are in line with reported ones in TRH knockouts of *Caenorhabditis elegans*, *Drosophila* and mice, indicating that there is one gene encoding TRH, which is the serotonin limiting enzyme in both the central and the periphery nervous system in *Daphnia* and that deprivation of serotonin increases anxiety-like behavior.

## Introduction

In arthropods, serotonin stimulates insect ecdysteroid and juvenile hormone production, both responsible for controlling oogenesis and vitellogenesis^1–3^. In decapod crustaceans, serotonin also stimulates growth- and reproduction-controlling hormone systems^4^ and serotonergic neurons are involved in control of behavior and metabolism^5–8^. However, in non-decapod crustacean species, such as *Daphnia*, we know little about the distribution and role of serotonin^9^, the latter only inferred from effects of exogenous exposures to selective serotonin reuptake inhibitor (SSRIs)^10,11^. *Daphnia* genome encodes serotonin biosynthesis enzymes, strongly suggesting the presence in *D. magna* of a serotonergic system, potentially involved in growth and reproduction. We previously found that individuals of *D. magna* exposed to SSRI fluoxetine and fluvoxamine mature earlier and produce more but smaller offspring^12,13^. Consistently, fluoxetine increases brain serotonin-immunoreactivity levels at low food conditions, bringing them to the maximal levels normally only observed under high food conditions, and enhancing offspring production concomitantly^14^. Sublethal amounts of the neurotoxin 5,7-dihydroxytryptamine, selectively killing serotonergic neurons, markedly decrease serotonin-immunofluorescence and offspring production in fluoxetine-treated animals, supporting that the observed effect on reproductive phenotype is modulated by serotonin levels^14^. Producing small clutches of larger offspring is an adaptive response to food scarcity in natural conditions, since the larger the offspring the more resistant to starvation^15^. This means that by increasing the levels of serotonin in the brain, SSRI produce maladaptive clutches of offspring. Physiological, transcriptomic, and behavioral studies also reported other side effects of exposure to SSRI including enhanced levels of oxidative and sugar metabolism, reduced tolerance to low oxygen concentrations and a positive phototactic behavior^12,16,17^. Diminished tolerance to low oxygen levels or having a positive phototactic behavior is maladaptive since in the field *Daphnia* migrates to deeper waters during the day to prevent fish predation and often these waters have low oxygen levels^18^. Therefore previous experimental studies suggest that serotonin activity may regulate the perception of food environment, which helps *Daphnia* individuals to have optimal metabolism, growth, reproduction, and behavior parameters across variable food environments. This premise, however, has only been indirectly tested using exposures to bio-active compounds (i.e. SSRI) and not by directly manipulating serotonin.

Pharmacological treatments are difficult to interpret because they are generally not specific and can have unwanted side effects on other amines. Moreover, their effects can vary according to the developmental period. Thus, genetic models offer a new way to explore the consequences of reduced serotonin in a more selective manner. Serotonin synthesis is mediated by the rate limiting enzyme tryptophan hydroxylase (TRH), which is encoded by one single gene form in *Caenorhabditis elegans*^19^. In *Drosophila melanogaster* and mammals there are two distinct and non-overlapping rate limiting enzymes for serotonin synthesis: TRH1 and TRH2 for peripheral nervous system (PNS) and central nervous system (CNS), respectively, in mammals; and tryptophan phenylalanine hydroxylase and TRH for PNS and CNS, respectively, in *D. melanogaster*^19,20^. Deletion of TRH-encoding genes in *C. elegans* results in fully viable animals completely deprived of serotonin. These mutants show alterations in their behavior and metabolism that are compatible with the sensation of food starvation: decreased feeding and offspring production rates and accumulation of large amounts of fat^21^. In *D. melanogaster*, TRH−/− null mutations lacking serotonin in the CNS but not in the PNS, only produce a moderate lethality during larval and pupae stages and induce mild effects on feeding or developmental rates^19^. Single TRH2−/− and double TRH1−/−/TRH2−/− knockout mice are viable and normal in appearance, only showing enhanced anxiety-like behavior^22^.

Our aim is to study the role of serotonin regulating growth, reproduction and behavioral responses in *D. magna*, by generating clonal lines bearing different mutations on the TRH gene enzyme. Using recently developed CRISPR-Cas9 genome editing tools for *D. magna*^23^, we obtained seven mutant lines: three bearing a mono-allelic indel TRH mutation (TRHA−/+, TRHB−/+, TRHC−/+) and having normal serotonin levels, and four bi-allelic indel (TRHA−/−; TRHB−/−, TRHC−/−, TRHD−/−) mutants completely deprived of serotonin. Life-history and behavioral studies under low and high food conditions and under exposure to the SSRI fluoxetine were conducted to test the hypothesis that TRH−/− lacking serotonin will have the reverse phenotype of SSRI exposed organisms: grow and reproduce less than wild type ones having an enhanced negative phototactic behavior.

## Results

### CRISPR-Cas9 mutants

**Figure 1:**
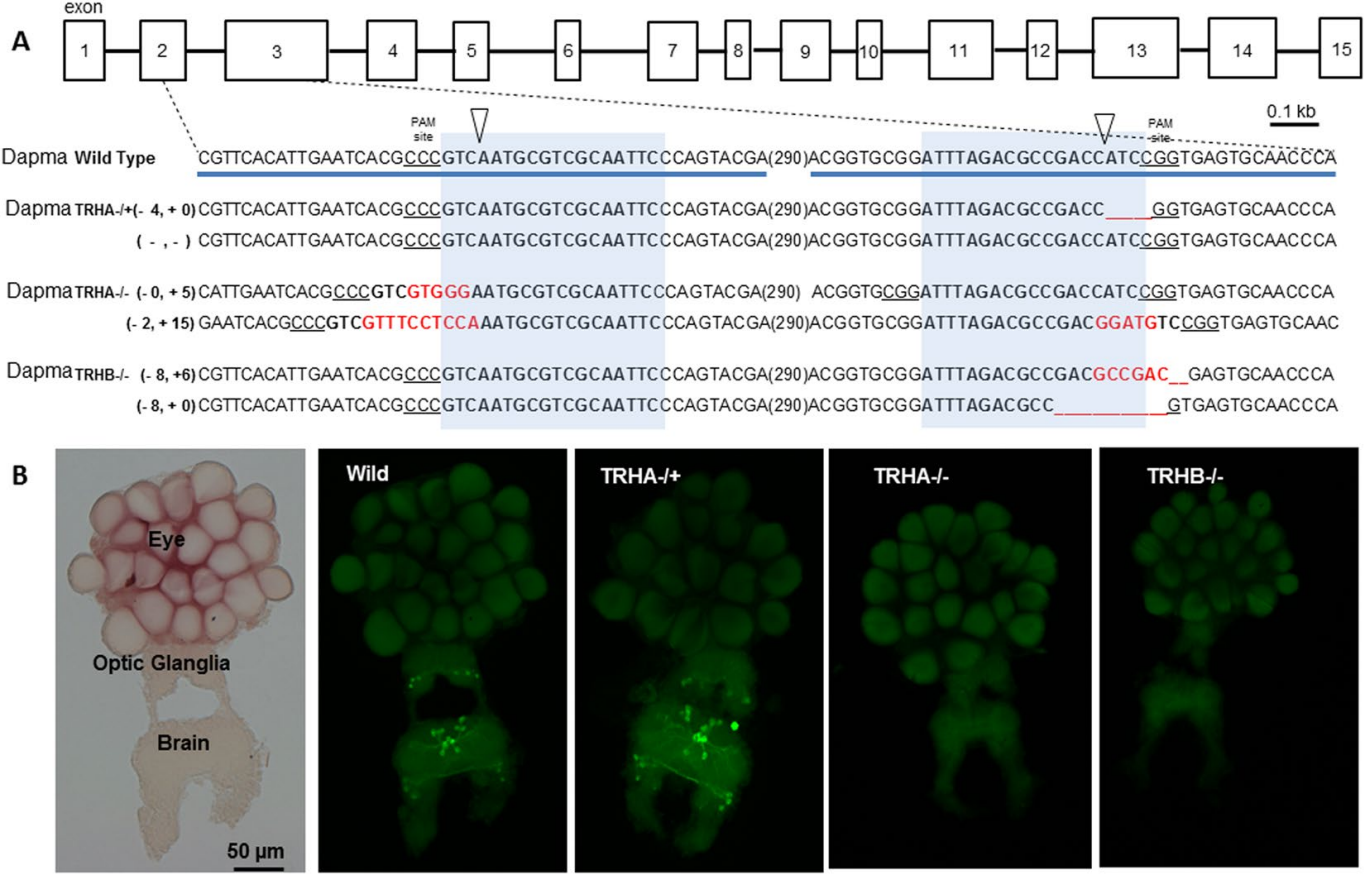
Knockout clones for the TRH gene lack serotonin immunofluorescence in the CNS. (**A**) Genomic sequences of three *DapmaTRH* knocked-out mutant lines in correspondence of gRNA-targeted sites. (**A**) part of the exon-intron structure of the *DapmaTRH* gene is shown above. The top line represents the wild-type *DapmaTRH* gene sequence (part of exon 2 and 3 are underlined) and subsequent lines show sequences of three mutant lines. The target sites for gRNAs are indicated in bold and blue, PAM sites are underlined and cleavage locations are shown by inversed triangles. The length (in base pairs) of each indel mutation is marked on the left of each sequence (− stands for deletions; + for insertions). In the sequences, deletions are indicated by underbars, insertions in red and the length of a truncated sequence within a parenthesis (in base pairs). (**A**) Obtained phenotypes for the serotoninergic system in the central nervous system (CNS) of *Daphnia magna*. From left to right images show a bright-field microphotograph of the CNS that includes the eye, medulla, and brain, and microphotograph of the CNS of representative wild-type (Wild), TRHA−/+ ,TRHA−/− and TRHB−/− after immunofluorescence against serotonin.

There is only one probable orthologue of mammalian tryptophan hydrolase in the *D. magna* genome (*Dapma7bEVm006764t1* hereafter referred as *DapmaTRH*, scaffold00084:361112-363778), which contains 15 exons (Fig. 1). Reverse transcription PCR of the *DapmaTRH* gene using a primer set encompassing the targeted mutagenic sites (Supplementary Methods, Table S1) as well as sequencing of the PCR fragments revealed that this gene is transcribed in *D. magna* (Supplementary Fig. S1). Mutations of the *Dapma THR* gene generated by CRISPR-Cas9 using the two target mRNA shown in Supplementary Methods, Table S2 produced 17 independent clones having indel mutations, as identified by PAGE analysis on the corresponding PCR-amplified DNA products (primers for these PCR-amplified products are in Supplementary Methods, Table S1**)**. Sequencing of seven of these clones detected mono-allelic indel *Dapma THR* gene mutations in three of them and bi-allelic indel ones in the other four. Figure 1A shows the sequence of the indel mutations present in clones TRHA−/+ , TRHA−/−, TRHB−/− (full *Dapma THR* sequences of the seven clones are in Supplementary Fig. S2). Brain inmunofluorescence analyses using an anti-serotonin in clones TRHA−/+ , TRHA−/−, TRHB−/− showed a total abolition of serotonin in the bi-allelic indel mutants, whereas mono-allelic indel mutants exhibited serotonin inmunofluorescence levels similar to the wild type control across the optic ganglia and brain (Fig. 1B).

**Figure 2:**
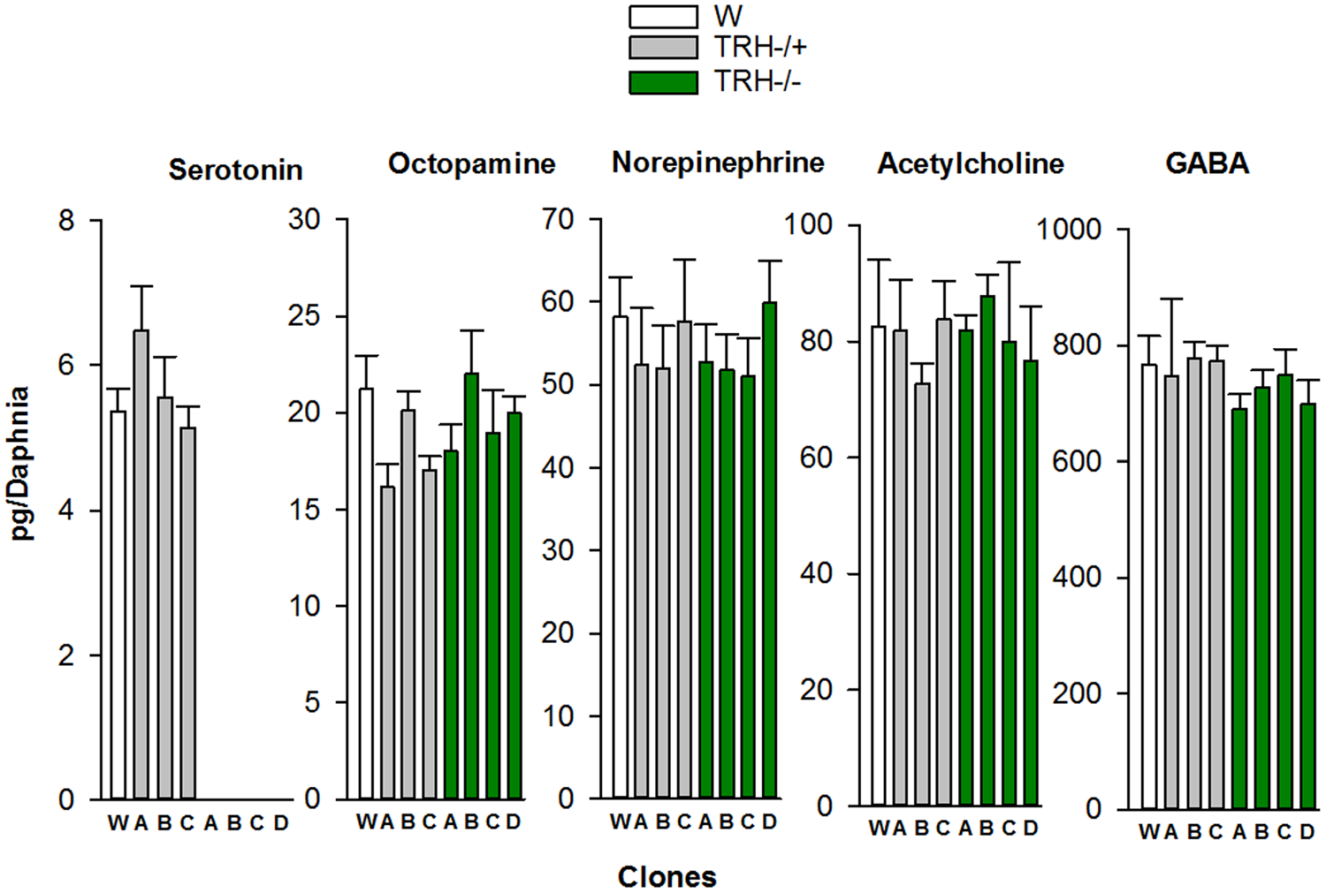
Bi-allelic indel mutated TRH clones lack serotonin in their whole body but have similar levels of other neurotransmitters. Residue levels (Mean ± SE, N = 5–8) of the studied neurotransmitters measured by LC-MS/MS in whole animals. Stats are in Supplementary Table S3.

Mass spectrometric analyses of whole body extracts showed serotonin levels below detection limits (<0.03 ng/mL) for THR (−/−) clones, whereas mono-allelic clones showed levels of serotonin similar to those of the wild type (Fig. 2, stats results are depicted in Supplementary Table S3). The results of Figs. 1B and 2 provide strong experimental evidence indicating that the TRH is the rate limiting enzyme for serotonin synthesis in both the CNS and the rest of the body. Measured tissue levels of octopamine, GABA, norepinephrine and acetylcholine in studied clones **(**Fig. 2) did not changed significantly (P < 0.05) (stat results are in Supplementary Table S3).

### Life history responses

**Figure 3:**
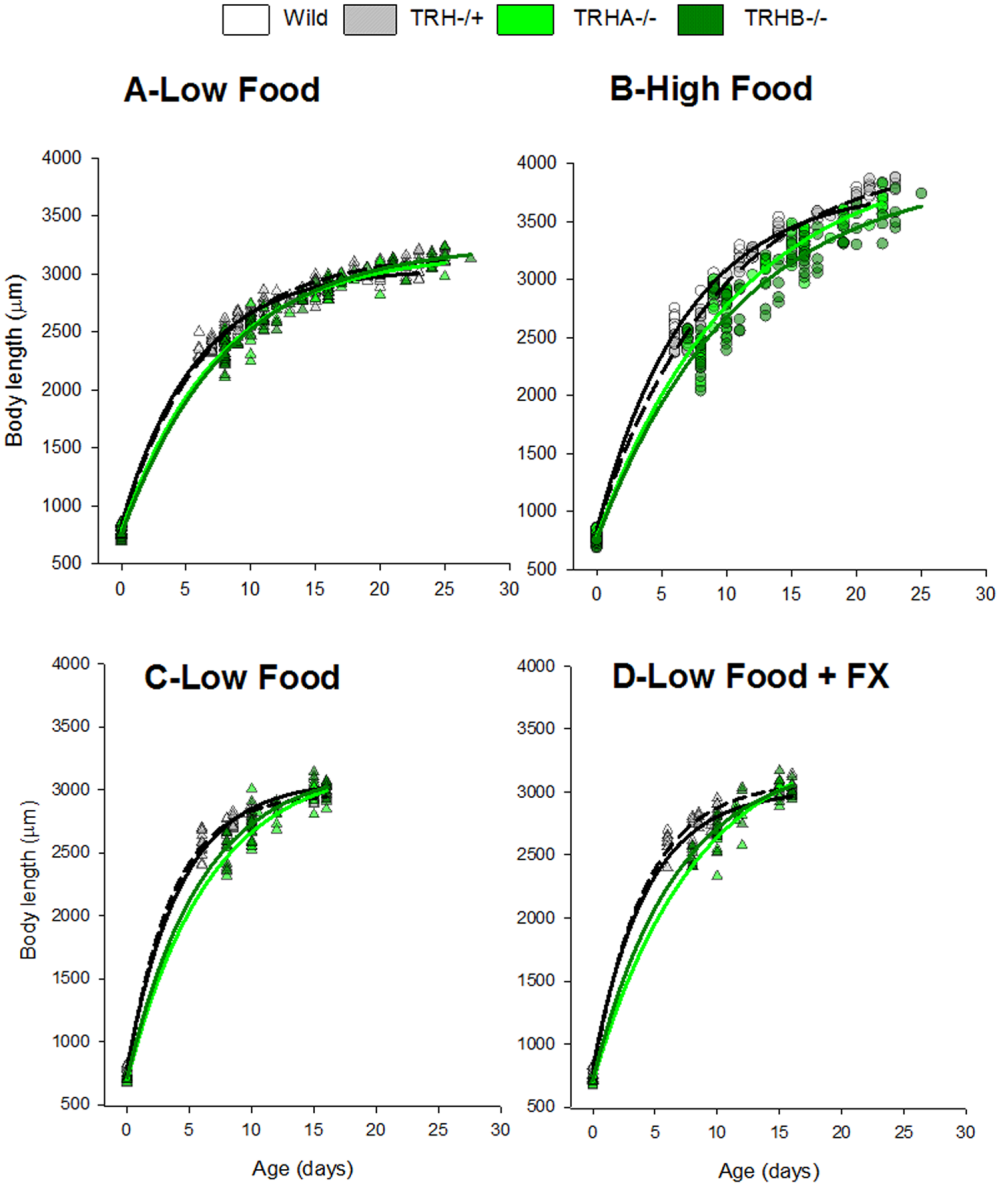
Bi-allelic indel mutated TRH clones lacking serotonin grew less than the mono-allelic mutated TRH and wild ones and fluoxetine did not affect growth. Body length measurements and fitted von Bertalanffy growth for *D. magna* individuals of the clones TRHA−/+, TRHA−/−, TRHB−/− cultured under low (**A**) and high (**B**) food ratios in the food study and of the same clones cultured at low food without (**C**) and with fluoxetine (FX, D) in the SSRI study. Continuous and dashed black lines correspond to fitted curves for wild and TRH−/+ clones, respectively, whereas green lines to TRH−/− ones. Stats are in Supplementary Table S4.

Body-length growth curves for clones TRHA−/+ , TRHA−/−, TRHB−/− cultured at low and high food rations in the food study (Fig. 3A,B) and exposed to fluoxetine (Fig. 3C,D) showed that TRH−/− clones lacking serotonin consistently grew less than the wild type clone and that fluoxetine did not affect the relative growth performance (Fig. 3, the full set of growth curves for the seven clones tested in the food experiment are in Supplementary Fig. S3). Clonal differences on grow rates were more evident at the high food ration since this food environment promoted to a greater extent *Daphnia* growth than low food. Clonal differences in size were also greater in females that just matured (6–8 days old) and released the first clutch of neonates (9–11 days old). This explains the observed significant (p < 0.05) effects of food ratio, clone and their interaction on body length measurements across adult states (full ANOVA results plus von Bertalanffy growth curve regression parameters are in Supplementary Table S4). In the SSRI experiment the growth performance of the four studied clones was quite similar without and with fluoxetine. Only individuals of clone TRHA−/+ showed a slightly greater growth relative to clone TRHB−/− under exposure to fluoxetine. This latter effect accounted for significant (P < 0.05) interaction terms between fluoxetine and clone or adult instar rates (full ANOVA results plus von Bertalanffy growth curve regression parameters are in Supplementary Table S4).

In the food study the lower growth of TRH−/− females resulted in the investment of more juvenile instars until maturity, in a delayed reproduction, and in a lower total offspring production and consequently lower population growth rates even at high food levels (Table 1, full set of results for the seven clones are in Supplementary Table S5). Nevertheless, differences in fecundity among clones became non-significant (P > 0.05) when female body size was considered as covariate in an ANCOVA (results and stats are shown in Supplementary Figure S5). In addition, TRH−/− females produced smaller offspring than their wild type counterparts (Table 1). The mono-allelic indel clones (TRH−/+ ) showed an intermediate phenotype between the wild type and TRH−/− clones. Neither TRH−/+ nor TRH−/− clones differ on their feeding and oxygen consumption rates or in the accumulation of storage lipids on lipid droplets relative to wild type clones (see stats and results in Supplementary Tables S3, S6, respectively). In the SSRI study fluoxetine despite of increasing cumulative fecundity did not affect the clonal relative responses of the studied life-history traits **(**Table 1**;** stats are in Supplementary Table S7).

**Table 1:** Life-history results from the food and SSRI studies showed reduced growth, offspring size and population growth rates, decreased fecundity at high food, and delayed reproduction in indel mutated TRH clones lacking serotonin. Fluoxetine despite of increasing cumulative fecundity did not affect the clonal relative responses.

	N	K (day-1)	N	MI (%)	N	Age (days)	N	Fec	N	Off Size (µm)	N	r (day-1)
**Food Study**												
W Low	105	0.175 ± 0.005	20	25	20	9.1 ± 0.1	19	21.9 ± 0.5	19	889.2 ± 4.5	20	0.259 ± 0.004
TRHA−/+ Low	101	0.152 ± 0.003	20	35	20	9.6 ± 0.2	20	22.9 ± 0.5	20	909 ± 9.6	20	0.261 ± 0.004
TRHA−/− Low	100	0.132 ± 0.005	20	100*	20	10.5 ± 0.2*	20	23.2 ± 0.4	18	857.5 ± 9.2*	20	0.244 ± 0.003*
TRHB−/− Low	96	0.122 ± 0.003	20	100*	20	11 ± 0.2*	20	23.7 ± 0.5	18	842 ± 9.8*	20	0.239 ± 0.003*
W High	92	0.143 ± 0.004	20	0	20	8.5 ± 0.1	20	48.3 ± 0.8	20	792.9 ± 10.6	20	0.342 ± 0.003
TRHA−/+ High	102	0.108 ± 0.003	20	30*	20	9.3 ± 0.2*	20	47.6 ± 1.4	20	793.9 ± 4.2	20	0.313 ± 0.002*
TRHA−/− High	105	0.092 ± 0.005	10	100*	10	9.7 ± 0.2*	20	41.2 ± 1.5*	20	763.5 ± 4.9*	10	0.296 ± 0.005*
TRHB−/− High	98	0.092 ± 0.006	18	100*	18	10.5 ± 0.3*	18	39.4 ± 1.3*	17	774.3 ± 4.9*	18	0.275 ± 0.008*
**SSRI Study**												
W Low	34	0.233 ± 0.01	9	56a	9	9.3 ± 0.3a	9	32.8 ± 0.7a	9	839.9 ± 7.3b	9	0.269 ± 0.007b
TRH−/+ Low	34	0.28 ± 0.014	10	30a	10	9 ± 0.2a	9	34.8 ± 0.6a	10	867.6 ± 5.0c	9	0.276 ± 0.006b
TRHA−/− Low	33	0.151 ± 0.018	9	100b	9	11.2 ± 0.3b	9	34.9 ± 0.8a	7	839 ± 4.8b	9	0.219 ± 0.007a
TRHB−/− Low	36	0.169 ± 0.012	10	100b	10	10.4 ± 0.3b	10	37.4 ± 0.6b	10	818.8 ± 7.6a	10	0.24 ± 0.006a
W Low + FX	32	0.235 ± 0.021	9	44a	9	9.1 ± 0.2a	9	37.2 ± 1.1b	8	860.6 ± 7c	9	0.273 ± 0.008b
TRH−/+ Low + FX	35	0.243 ± 0.013	10	50a	10	9.2 ± 0.2a	10	37.8 ± 1bc	10	867.4 ± 5.2c	10	0.272 ± 0.006b
TRHA−/− Low + FX	34	0.121 ± 0.014	9	100b	9	11.4 ± 0.2b	9	40.3 ± 1c	8	812.8 ± 3.2a	9	0.222 ± 0.004a
TRHB−/− Low + FX	35	0.152 ± 0.011	10	100b	10	10.6 ± 0.3b	10	41.1 ± 0.8c	10	805.8 ± 3.1a	10	0.242 ± 0.006a

Mean vales (±SE) for the von Bertalanffy grow rate parameter (K), percentage of individuals investing more than five juvenile instars to maturity (MI, first releasing of eggs into the brood pouch), age at first reproduction, cumulative offspring production of the first three broods (Fec), offspring size (Off size), population growth rates (r) of *D. magna* individuals from the studied clones. In the Food Study within a given food *indicates significant (p < 0.05) differences from the wild type clone following ANOVA and Dunnett’s or the equivalent non parametric post-hoc comparison tests. In the SSRI study different letters indicated significant (p < 0.05) differences from the wild type clone following ANOVA and Tukey’s or the equivalent non parametric post-hoc comparison tests. Stats are depicted in Tables S3, 7.

**Figure 4:**
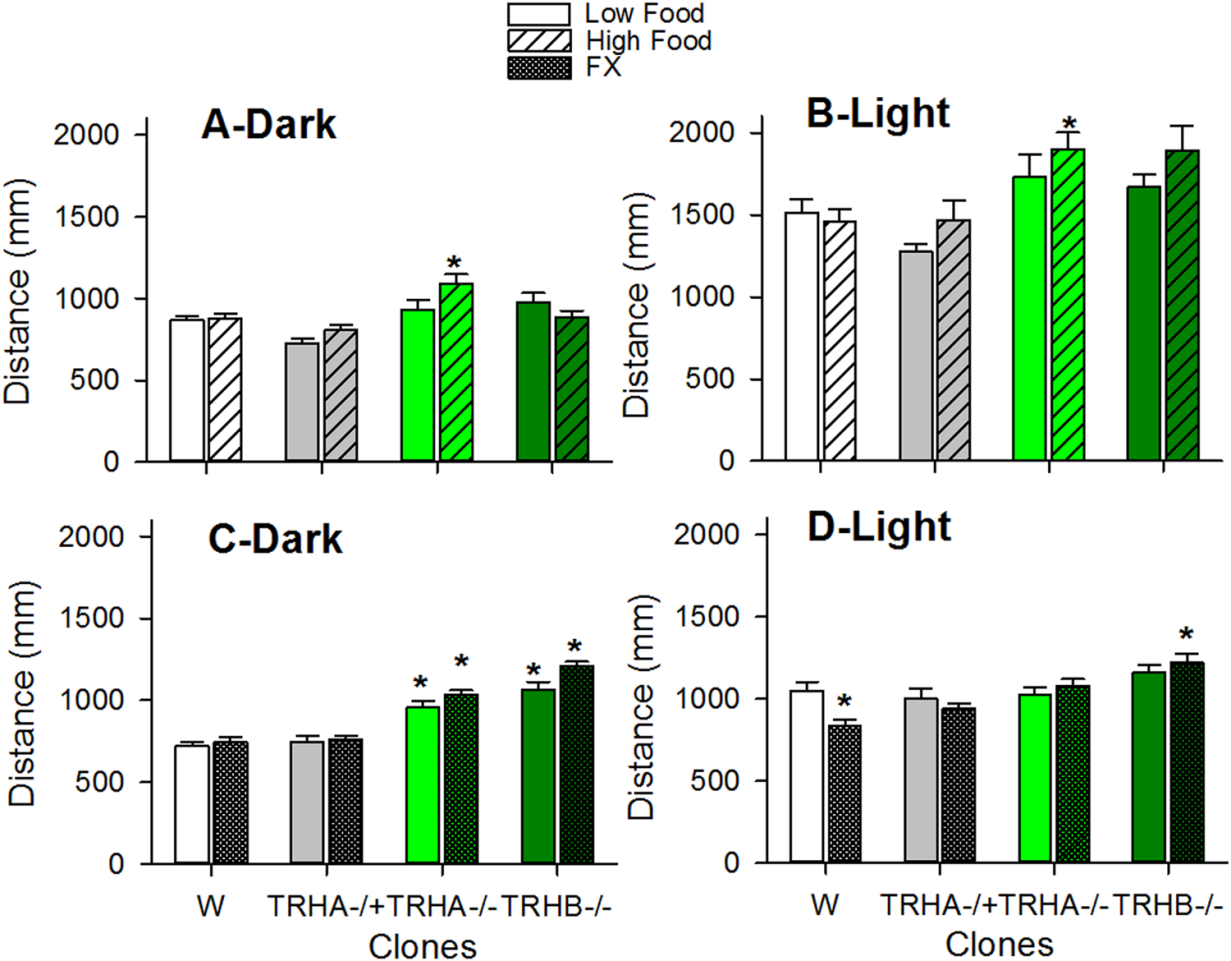
Bi-allelic indel mutated TRH clones lacking serotonin had an enhanced locomotor activity and responder more to light than the mono-allelic indel mutated TRH and wild ones. Fluoxetine decreased the response to light only in the wild type and the mono-allelic indel mutated TRH clones. Distance moved under dark (**A,C**) and light (**B,D**) conditions of the studied clones cultured under low and high food ration levels (**A,B**) or at low food levels and exposed to fluoxetine (**C,D**). Within a food environment and photoperiod *indicates significant (p < 0.05) differences from the wild type clone following ANOVA and Dunnett’s. Stats are depicted in Supplementary Tables S3, 7.

In the food study light naturally increases the swimming activity of *D. magna* individuals, a behavioral response that became significantly stronger in bi-allelic indel TRH−/− mutants relative to wild type or TRH−/+ clones (Fig. 4A,B). These effects were still more evident when exposure to light was combined with high food ratio levels (Fig. 4A,B, three-way ANOVA analysis in Supplementary Table S3). In the SSRI study the swimming activity of mutants lacking serotonin (TRH−/−) was greater than those having it, irrespectively of fluoxetine exposure (TRH−/+ , Wild type, Fig. 4C). The response to light of wild type individuals and to a lesser extent of those from the TRHA−/+ clone decreased under exposure to fluoxetine, whereas that of organisms from TRH−/− clones was unaffected (Fig. 4D). The above mentioned responses accounted for the observed significant interaction terms between fluoxetine, photoperiod and clone (three-way ANOVA analysis results are in Supplementary Table S7).

## Discussion

The CRISPR-Cas9 methodology has proved to be a powerful tool to introduce reverse genetics in many species previously considered as inaccessible, including humans.

In this paper, we used this approach to deepen on the study of the role of serotonin in *D. magna* reproductive and metabolic strategies. Furthermore using TRH knockouts lacking serotonin we also tested the premise that previously reported effects of the SSRI fluoxetine on reproductive and behavioral responses^12,14,16^ in *D. magna* were related to serotonin changes. CRISPR-Cas9 mutated, TRH (−/−) clones showed no detectable levels of serotonin, tested both by serotonin inmunofluorescence in neurons of optica ganglia and brain, and by direct serotonin quantification in the whole animal. More interestingly, the serotonin deprivation resulted in a specular counterpart of the complex behavior and biochemical phenotype observed in *D. magna* when exposed to SSRI, which results in and increased active concentration of serotonin in the brain.

The use CRISPR-Cas9 methodology in *D. magna* has been developed only recently, in part due to our relatively late knowledge of the *D. magna* genome, which has been fully decoded and annotated since 2016^24^, and in part because *D. magna* is a cyclical parthenogenetic species whose laboratory lines are maintained exclusively by asexual reproduction. Therefore, recessive phenotypes need to target both chromosomal alleles independently, and there is no actual homozygotic lines, but they have to be referred to as bi-allelic indel mutants. Our procedure obtained both mono- and bi-allelic indel mutants (TRH−/+ and TRH−/−, respectively); the fact that TRH (−/−) clones show no serotonin whereas TRH−/+ presented a concentration similar to the one observed in wild type clones demonstrated that *DapmaTRH* is the only gene encoding tryptophan hydroxylase, which is the serotonin limiting enzyme in both the CNS and the periphery. The finding that bi-allelic indel mutants lack serotonin agrees with previous results obtained in null TRH mutants of *C. elegans*^21^, but not with those reported in *D. melanogaster* neither in mammalian models, where two distinct enzymes synthetize serotonin in the CNS and periphery^19,20^. In *D. melanogaster* TRH synthetizes serotonin in the CNS whereas the tryptophan phenylalanine hydroxylase enzyme (*Dmel\Hn*) do so in peripheral tissues. In the *D. magna* genome there are two enzyme/genes, phenylalanine-4-hydroxylase 2 (*Dapma7bEVm002697*) and tyrosine 3-monooxygenase (*Dapma7bEVm009554*) showing 69 and 47% identity on their amino acid sequences with that of *Dmel\Hn*, respectively. This means that these gene/enzymes orthologue to the *Drosophila* Dmel\Hn may also work for serotonin production despite their lower contribution. The function of these gene/enzymes could be tested using Cas9-mediated knockout in the future. Measured tissue levels of octopamine, GABA, norepinephrine and acetylcholine were similar across the studied clones and were also within the range reported for other vertebrate and invertebrate species^25–28^, indicating no crosstalk between TRH and other neurotransmitter synthetizing enzymes. Nakanishi, *et al*.^23^ using CRISPR-Cas9 mediated targeted mutagenesis on a functionally conserved regulator of eye development, the eyeless gene in *D. magna*, found that all of the deformed eye individuals had bi-allelic indel mutations around gRNA-targeted sites, whereas mono-allelic indel mutations were found in normal eye individuals. The new LC-MS/MS analytical method developed to determine serotonin levels on whole *Daphnia* tissues had a detection limit of 0.03 ng/mL (Supplementary Table S9) equivalent to 0.2 pg/individual, which is comparable to other reported studies^25,26,29^, and tenfold lower than the observed levels in the wild type clone. Nevertheless future investigation should be focus in the application of the MALDI imaging mass spectrometry to allow determination the spatial localization and molecular distribution of metabolites and neurotransmitters within biological organisms^30^.

Serotonin is required and has a vital role in early embryogenesis in several species and it regulates many physiological processes including energetic metabolism, growth, reproduction and behavior in several species. Nevertheless, mice or *C. elegans* lacking serotonin or flies (*D. melanogasters*) having diminished levels of neuronal serotonin are viable and fertile^19–21^, suggesting that serotonin may modulate many behaviors and physiological processes, but is not the principal neurotransmitter for any of them. In the present study the lack of serotonin in TRH(−/−) clones did not affect embryo, juvenile or adult survival, but diminished growth rates, the size at birth and increased motor activity and the response to light. The combination of being smaller at birth and having lower growth rates ends up with the investment in more juvenile instars, which translated into delayed reproduction. This is in line with reported energy-based and mechanistic models for *Daphnia* growth and reproduction^31^. At high food levels being smaller and having lower growth rates resulted also in smaller reproductive females and the production of smaller clutches. At low food levels, delayed reproduction not necessary translated into smaller females and smaller clutches since these females took advantage of having an additional instar to grow. The above mentioned body size and reproductive effects are in line with the maturation size threshold hypothesis for *Daphnia* growth and reproduction, which translated into lower fitness measured as population growth rates^15,32^. Furthermore, TRH−/+ mutants, which apparently have wild-type levels of serotonin, showed reduced growth rates, albeit to a lesser extent than TRH−/− clones. This suggests that a single functional DapmaTHR gene is not enough for producing all the amount of serotonin required in at least some stages of the complex life cycle of *D. magna*, and it may indicate that THR is the rate-limiting step in the synthesis of serotonin in *D. magna*. Unfortunately in this study we did not succeed in measuring serotonin in early life stages of *D. magna* development and hence we cannot ruled out the hypothesis that mono-allelic indel TRH mutants may have lower levels of serotonin during early life stages, which may affect growth rates.

In *C. elegans* serotonin also modulates food perception but in a different way as it does in *Daphnia*. *C. elegans* null TRH mutants lacking serotonin showed abnormalities in behavior and metabolism related with starvation, which translated into lower fecundity and feeding rates and an enhanced accumulation of storage lipids reserves to cope with future harsh conditions^21^. In our study the lack of serotonin did not affect feeding rates, storage lipid accumulation or oxygen consumption rates. *Daphnia* growth and reproduction is tightly regulated by resource food acquisition, which depends on feeding rates^33^, that are regulated by food concentration^34^. *Daphnia* accumulates storage lipids into lipid droplets to use them for the formation of the new carapace and egg provisioning^35^. The amount of lipid droplets also varies with food and is regulated by molt and juvenile hormone receptors^35^. Oxygen consumption without food is a measure of basal metabolism^36^. In a previous work we found that SSRI increased serotonin immunoreactivity, basal oxygen consumption rates but not feeding nor lipids^12^. Regardless of oxygen consumption, thus, our results are in line with those reported previously in *Daphnia*^12^, indicating that serotonin effects on growth are independent of food acquisition and of basal and lipid metabolism.

In *D. melanogaster*, reported effects on serotonin synthesis mutants are more difficult to interpret since the complete abolition of serotonin is lethal. Null TRH flies having diminished levels of neuronal serotonin show only slightly reduced feeding rates, mild developmental delays, and display some lethality during larval stages, with no significant size defects^19^. Interestingly, *Dmel\Hn* knockdown larvae, which lack peripheral serotonin, show hyperactivity (enhanced movement) whereas null TRH adult flies display a diminished locomotor behavior^19^. In our study null TRH *D. magna* mutants increased locomotion, consistently with the behavior of *D. melanogaster* knockouts for periphery serotonin synthesis. Double knockouts TRH1/TRH2 mice, completely deprived of serotonin, are apparently normal in appearance but show an increased anxiety-like behavior^22^, again in line with our behavioral results. *D. magna* clones that evolved in environments having fish develop a marked negative phototactic behavior to avoid fish predation^37^. In our behavioral setup, wild type *D. magna* individuals responded to light increasing their motor responses and hence swimming longer distances. Null TRH individuals not only moved more but also responded to a greater extent to light stimuli, a behavior that can be related to the known association between higher swimming activity under light and anxiety in amphipods^38^.

Exposure to the SSRI fluoxetine provide evidence that behavioral effects of this drug in *D. magna* may be associated to serotonin but reproductive effects do not. Fluoxetine despite of increasing cumulative fecundity did not affect the clonal relative life-history performance of TRH knockout clones lacking serotonin. Conversely fluoxetine decreased the response to light stimuli only in individuals having serotonin. The previous results indicate that fluoxetine may have multiple modes of action in *D. magna*. In the nematode *C. elegans*^39^ fluoxetine and other antidepressant (imipramide) act throughout different mechanisms at the serotonin receptors. Even *C.elegans* mutants completely lacking serotonin like our TRH−/− clones respond to these drugs. More specifically fluoxetine can stimulates egg laying in *C.elegans* (a well-established serotonergic response) activating a Gq protein in mutants lacking serotonin or/and its associated receptors^39^. Thus a similar mechanism may exist in *D. magna*. Future research on gene signalling pathways should be performed to elucidate putative mechanisms of action of fluoxetine.

Observed effects of fluoxetine on behavior, however, agree with previous reported effects in *Daphnia* and amphipod crustaceans. In the absence of light individuals of clones lacking serotonin moved more and fluoxetine did not affect locomotion activity in any of the studied clones. Upon exposure to light fluoxetine diminished the locomotion activity of individuals having serotonin but did not affect those of knockout clones lacking it. Rivetti *et al*.^16^ found that fluoxetine at the concentrations tested here decreased the negative response to light in *D. magna* individuals, and similar results were reported in the marine amphipod *Echinogammarus marinus* exposed to fluoxetine or serotonin^40^. Therefore our results and those previousy reported in *Daphni*a and in amphipods support the argument that serotonin regulates the phototactic behavior. Nevertheless, the mechanisms by which the serotoninergic system regulates behavior is complex in arthropods. In *D. melanogaster*, dysregulation of serotoninergic system with either too much or too little serotonin decrease locomotion activity due to the existence of compensatory mechanisms associated to the involvement of different serotonin receptors having antagonistic effects^41^. This means that the serotoninergic system that regulates behavior is complex and deserves to be exhaustively studied using genetic and exogenous manipulations.

Reported effects of serotonin in crustaceans is almost limited to pharmacological studies in decapods^4^. Exposure to serotonin or serotonergic drugs increased the levels of the crustacean hyperglycaemic hormone, which regulates energy metabolism^7^, stimulated ovarian maturation^42^ and anxiety-like and aggressive behavior in decapods^6,8^. Only the reported stimulatory effects of serotonin on decapod reproduction agrees with the expected phenotype of *D. magna* individuals deprived of serotonin. *D. magna* individuals lacking serotonin reproduced less and did not show any metabolic effect neither a reduced anxiety like behavior. *Daphnia* physiology and behavior share insect- and decapod crustacean-like features. Genomic, transcriptomic and peptidomis studies show that *Daphnia* neuropeptides that can be affected by serotonin are phylogenically close to insects^43–46^, whereas some key hormones such as methyl farnesoate are closely related to decapods^47^. There is also no evidence of aggressive mating or territorial behavior in *Daphnia* probably due to its planktonic mode of life^18^. Therefore, our findings are consistent with a close relationship of *Daphnia* and insects, which has been inferred from several molecular, genomic and physiological studies^46,48,49^.

Recently there are several reports indicating that in cultured cells and mice, CRISPR-Cas9 off-target mutations are high, but in zebrafish and *Drosophil*a, are low^50–53^. Phylogenetically *D. magna* genome is quite close to that of *Drosophila* and our insilico check using BLASTn (see Supplementary Methods) did not find any potential off-target sites. Furthermore, knockout resulted in reproducible phenotypes that should be observed in TRH−/− mutant based on our previous studies^12,14^: (1) among the neurotransmitters analyzed, only serotonin was not detected, and the serotonin deprivation resulted in a specular counterpart of the complex behavior and biochemical phenotype observed in *D. magna* when exposed to SSRI inhibitors, which results in and increased active concentration of serotonin in the brain, (2) mono-allelic indel TRH−/+ individuals were hypomorph showing the intermediate phenotype, indicating dosage-dependent phenotypes. Taken together the above mentioned effects, we are sure that target mutations at the TRH locus had major impacts on phenotypes of mutants although there is a possibility that off-target mutations are present and might have small effects on the phenotypes we observed.

In summary, our results indicate that TRH is apparently the only enzyme for serotonin biosynthesis in *D. magna*. The lack of serotonin in bi-allelic indel mutants reduced growth rates and offspring size independently of food, and increase the response to light. Obtained results were consistent across experiments and changed across food levels in an expected way, as individuals grew less and produced bigger offspring at low food than at high food conditions. Exposures to the SSRI fluoxetine despite of increasing cumulative fecundity did not affect the clonal relative life-history responses, which means that this drug affects reproduction independently of serotonin. Fluoxetine did affect behavioral responses in an expected way, decreasing the response to light only in those individuals having serotonin. The other observed life-history changes (reproduction, population growth rates) were related with growth-mediated effects on mother’s body size and time to first reproduction. The molecular mechanisms by which serotonin regulates growth and fluoxetine reproduction in *Daphnia* are still unclear. In both *C. elegans* and *Drosophila*, serotonin and insulin jointly control growth^21,54,55^. In *C. elegans* fluoxetine can stimulates egg laying activating a Gq protein, which is located downstream the serotoninergic signalling pathway^39^. *Daphnia* genome includes many of the genes and peptides involved in the *Drosophila* and *C. elegans* insulin and serotoninergic signalling pathway^46,56,57^, but little is known on their physiological role^46^. Further research would decide whether the insulin/serotonin axis is responsible for the observed reproductive, growth, and behavioral effects observed in *Daphnia* upon disruption of serotonin function either by genetic abolition of its biosynthesis or by chemical interference in its metabolism and/or signalling pathway.

## Methods

### Experimental animals

A single *D. magna* clone F, extensively characterized in previous studies^58^, hereafter designated as wild type clone (W), were used as the source for generating the seven studied CRISPR-Cas9 mediated TRH mutant clonal lines, three TRH−/+ mono-allelic indel TRH mutants and four TRH−/− bi-allelic indel mutants.

For each clone individual or bulk cultures of 10 animals/l were maintained in ASTM hard synthetic water as it has been described previously^33^. Individual or bulk cultures were fed daily with *Chorella vulgaris* Beijerinck (5 × 10^5^ cells/ml, corresponding to 1.8 μg C/ml^33^). The culture medium was changed every other day, and neonates were removed within 24 h. Photoperiod was set to 16 h light: 8 h dark cycle and temperature at 20 ± 1 °C.

### CRISPR-Cas9 mediated targeted mutagenesis

The sequence of the probably single orthologue of the mammalian tryptophan hydrolase in the *D. magna* genome (*Dapma7bEVm006764t1* hereafter referred as *DapmaTRH*, scaffold00084:361112-363778) was obtained from the *D. magna* genome consortium (www.http://wfleabase.org). After confirming the presence of the corresponding THR mRNA in the *D. magna* transcriptome by Reverse Transcription-PCR and amplicon sequencing using the primers of Table S1 (Supplementary Fig. S1), CRISPR-Cas9 targeted mutagenesis was performed according to Nakanishi, *et al*.^23^ (full description is in Supplementary Methods).

### Experimental design

Two different studies were conducted: a food study aimed to determine the life-history and behavioral performance of the studied seven mutant clones across low and high food levels, and a SSRI study whose objective was to test the effects of fluoxetine on life-history and behavioral responses in selected mutated clones having and lacking serotonin. This will allow to confirm our previous findings that fluoxetine affects reproduction and behavior in *Daphnia* enhancing brain serotonin activity^14^.

### Reproduction

Effects on reproduction were assessed followed established OECD guidelines with only minor modifications^58^. The food study was conducted with the selected seven TRH mutated clones plus the wild type one (W). Two independent an almost identical experiments were performed. The first experiment included the seven studied mutated clones plus the wild type one and aimed to determine life-history effects. The second one was performed with clones TRHA−/+, TRHA−/−, TRHB−/− and the wild type one and aimed to study how consistent were measured life-history and behavioral effects. In the first and second experiment neonates (<24 h old) were exposed until their fifth and third brood, respectively, at low and high food ratio conditions (1 and 5 × 10^5^ cells/ml of *C. vulgaris, respectively*) at 20 °C. Animals were exposed individually in 100 mL of ASTM hard water. Each treatment was replicated 10 times. The test medium was changed every other day. Measured life-history traits were: initial and body length of experimental animals across adult stages; juvenile and adult survival; age at first reproduction; clutch size and population growth rates (r), estimated from the age specific survival and reproduction rates according to the Lotka equation^33^. Body length measurements were performed from the top of the head to the base of the spine using a Nikon stereoscope microscope (SMZ 150, Nikon, Barcelona, Spain) and the ImageJ software (http://rsb.info.nih.gov/ij/). Clonal growth length curves across food ration levels were estimated from body size measurements of experimental animals by non linear least-squares fit to the von Bertalanffy function^59^:

1 $${L}_{t}={L}_{max}(1-{e}^{-K(t-t0)})$$

where Lt is body length, L_max_ is asymptotic length, k is the growth rate, t is age and t0 is included to adjust the equation for the initial size of the organism and is defined as age at which the organisms would have had zero size. In each case, residuals of the least-squares models were examined to assess goodness-of-fit and possible bias. Despite that *Daphnia* growth is stepwise the von Bertalanffy function take into account age and initial length at birth length, which may help to correctly interpret observed differences in body size across the studied clones. In the SSRI study four clones TRHA−/+, TRHA−/− and TRHB−/− and the wild type one were exposed to 100 ng/L of fluoxetine (dissolved in water) at low food level (1 × 10^5^ cells/ml of *C. vulgaris)* since our previous results showed that at low food levels effects of fluoxetine enhancing reproduction were the greatest^12–14^. Treatments included unexposed and exposed treatments for each clone replicated 10 times. Animals were exposed individually from birth until the third brood and their growth and reproduction responses measured. The remaining experimental conditions were similar to the “food study”. We selected 100 ng/L since it is an environmental relevant concentration of fluoxetine that can be found in surface waters^60^.

### Phototactic Behavior

Changes in phototactic behavior were limited to the wild clone (W) and three knockout clones TRHA−/+, TRHA−/− and TRHB−/− and were analysed by measuring the distance moved by first brood females. In the food supply study animals from experiments 1 and 2 were used. In the SSRI study an additional experiment was performed whose experimental conditions were identical as those depicted for reproductive responses but treatments were replicated 20 times. Measurements were conducted placing individual females in 24 well-plates filled with the cultured media. The plate was transferred into the behavioral testing chamber equipped with a temperature control unit (DanioVision, Noldus Information Technology, Leesburg, VA). Animals were then acclimated in the dark for 30 min before video recording. The video tracking conditions used consisted on 8 min cycle including a first 2 min dark period followed by a first 2 min light period, a second 2 min dark period and a second 2 min light period. The position of each individual daphnia was recorded using an IR digital video camera Basler acA1300-60gm (Basler Inc., Exton, PA) and an EthoVision XT 11.5 video tracking system (Noldus Information Technology, Leesburg, VA). Tracks were analyzed for total distance moved (mm) calculated for each dark or light period. All microplates were analyzed at 20 ± 0.5 °C with same detection and acquisition settings. Recently a similar approach was used successfully to detect changes in phototactic behavior of amphipods exposed to Prozac^38^.

### Physiological effects

Clonal differences on feeding rates, oxygen consumption rates and storage lipid accumulation were limited to the wild, TRHA−/+ , TRHA−/− and TRHB−/− clones whereas brain immunofluorescence and the levels of neurotransmitters were determined in the seven studied clones plus the wild type one. Feeding rates, oxygen consumption and neurotransmitters were studied in forth instar juveniles (4–5 day old animals at 20 °C), whereas storage lipid accumulation and brain serotonin immunofluorescence were assessed in first brood females. Two independent experiments spaced in time were performed for each determination. All experiments were conducted at high food ration conditions. Experiments were initiated with newborn neonates <12 h old, which were reared in groups of five in 100 ml of media until the end of the forth juvenile instar and then cultured individually until the release of the first clutch of eggs into the brood pouch. The test medium was renewed every other day. Experimental procedures for determining feeding, oxygen consumption and storage lipid accumulation in lipid droplets followed previous methods^35,58,61^ that are fully described in Supplementary Methods.

### Brain whole-mount immunofluorescence

Methods for immunofluorescence microscopy were performed as described previously^14^ and are described in described in Supplementary Methods.

### Neurotransmitter analysis

Analysis of neurotransmitters in *D. magna* whole body extracts was performed by liquid chromatography-tandem mass spectrometry following Tufi, *et al*.^29^ with minor modifications. The description of the analytical method is provided in Supplementary Methods.

### Data analyses

Levels of neurotransmitters, feeding, oxygen consumption and lipid droplets measured in the wild type clone and in mono and bi-allelic indel mutants were compared by one way ANOVA. Life history responses of the studied clones within and across food levels and fluoxetine treatments were compared by means of two way ANOVA analyses and behavioral ones by three way ANOVA considering light regimen (light or darkness) as an additional factor. The body length of females was measured across consecutive adult instars thus a repeated measurement ANOVA was selected considered adult instar as a repeated variable and food and clone or fluoxetine treatment and clone as factors. Within each food level the effect of the covariate maternal body length on clutch size responses among clones was also tested using ANCOVA. Prior to analysis data was checked to meet ANOVA assumptions of normality and variance homoscedasticity^62^. Age at first reproduction and the percentage of females maturing at five or more instars did not meet ANOVA assumptions thus were analyses respectively by Kruskal-Wallis, Mann-Whitney and Chi-square tests. Following analyses post hoc Dunnett’s, Tukey´s or the non-parametric equivalent tests were used to compare indel mutated clones against the wild type one or all clones^62^. Each analysis for the food study included data from two independent experiments.

## Electronic supplementary material

Supplementary Information
