# Supplementary material for: Tryptophan hydroxylase (TRH) loss of function mutations induce growth and behavioral defects in Daphnia magna
Source: Sci Rep. 2018 Jan 24;8:1518. doi: 10.1038/s41598-018-19778-0 (PMC5784079; doi:10.1038/s41598-018-19778-0)
Supplement: Supplementary file 1 — Supplementary Information [file 41598_2018_19778_MOESM1_ESM.pdf]

SUPPLEMENTARY INFORMATION

Tryptophan hydroxylase (TRH) loss of function mutations induce growth and behavioral defects in *Daphnia magna*

Claudia Rivetti, Bruno Campos, Benjamín Piña, Demetrio Raldua, Yasuhiko Kato, Hajime Watanabe, Carlos Barata

Supplementary Tables

Table S1. Oligonucleotides used for Dapma*TRH* **TRH transcript template and for the verification of cutting sites TRH-1, TRH-2, by sequencing**.

Name	Forward	Reverse
TRH transcript	GCGAAACGAGTGAACGTC	TGGAGAGCCTATTCCGAGAGT
TRH-1	GCGAAACGAGTGAACGTC	GCTGTTAAACTGGCCGAATT
TRH-2	CATCTCCGAATTGGACCAG	GGATTCAAAGACCCCGTGTA

Table S2 Oligonucleotides for mRNA gRNAs

gRNAs	Target	Forward	Reverse
TRH-1	GTCAATGCGTCGCAATTC	TAGGGAATTGCGACGCATTGAC	AAACGTCAATGCGTCGCAATTC
TRH-2	ATTTAGACGCCGACCATC	TAGGATTTAGACGCCGACCATC	AAACGATGGTCGGCGTCTAAAT

Supplementary Table S3. **Statistical results for the food study, neurotransmitters and physiological responses**.

	Factor	df	X^2	p
Maturation	CL	7	73.6	<0.001
	CH	7	97.3	<0.001
Age	CL	7	53.8	<0.001
	CH	7	70.5	<0.001
	Factor	df	F	
Fecundity	F	1,221	1118.8	<0.001
	C	7,221	7.6	<0.001
	F x C	7,221	8.8	<0.001
Offsring size	F	1,212	519.8	<0.001
	C	7,212	12.1	<0.001
	F x C	7,212	6.5	<0.001
r	F	1,223	221.9	<0.001
	C	7,223	42.4	<0.001
	F x C	7,223	11.0	<0.001
Swimming*	Ph	1,239	339.8	<0.001
	F	1,239	4.9	0.026
	C	3,239	16.5	<0.001
	Ph x F	1,239	1.4	0.237
	Ph x C	3,239	2.7	0.044
	F x C	3,239	1.2	0.309
	Ph x F x C	3,239	1.1	0.349
Serotonin**	CH	3,17	1.7*	0.194
Octopamine	CH	7,35	1.6	0.162
Norepinehrine	CH	7,35	0.5	0.791
Acetylcholine	CH	7,35	0.9	0.539
GABA	CH	7,35	0.3	0.942
Feeding*	CH	3,55	2.4	0.073
Oxygen*	CH	3,35	2.7	0.058
Lipid*	CH	3,66	0.2	0.924

Chi square and Kruskal-Wallis results testing for effects of clone (C) within each food ratio on the % of individuals investing more than 5 juvenile instars to maturity (Maturation) and age at first reproduction, respectively. ANOVA results testing for: the effects of food (F) and clone on total offspring production (fecundity), offspring size and population growth rates (r); for effects of photoperiod (Ph), food and clone on swimming distance (swimming); for effects of clone on detected levels of neurotransmitters, feeding responses, oxygen consumption rates and lipid droplets at high food ratio. df, $\chi 2$, F, p are degrees of freedom, Chi-square, Fisher's coefficient and probability level, respectively. L, H are low and high food ratios, respectively. * Analyses were limited to TRHA-/+, TRHB-/-, TRHC-/- and Wild type clones ** only TRH-/+ and wild type clones were considered.

Supplementary Table S4 . Growth results in the food and SSRI studies showed reduced growth in bi-allelic indel mutated TRH clones lacking serotonin and negligible effects of fluoxetine.

	von Bertalanffy regression					ANOVA			
Clone x Food	Lmax	K	t0	r2	N	Factor	df	F	P
**Food Study**									
W Low	3046.2±16.8	0.175±0.005	-1.83±0.06	0.99	105	I	1,221	7146.7	<0.001
TRHA-/+ Low	3175.1±16.2	0.152±0.003	-2.04±0.06	0.99	101	F	1,221	517.4	<0.001
TRHB-/+ Low	3104.8±20.8	0.178±0.007	-1.7±0.1	0.988	70	C	7,221	14.3	<0.001
TRHC-/+ Low	2967.3±28.5	0.151±0.007	-2.3±0.2	0.987	70	IxF	1,221	363.9	<0.001
TRHA-/- Low	3183.8±29.1	0.132±0.005	-2.13±0.1	0.99	100	IxC	7,221	7.1	<0.001
TRHB-/- Low	3259.1±24.7	0.122±0.003	-2.13±0.07	0.99	96	FxC	7,221	11.1	<0.001
TRHC-/- Low	3148.2±22.9	0.146±0.005	-1.9±0.1	0.968	68	IxFxC	7,221	2.2	0.036
TRHD-/- Low	3197.7±27	0.127±0.004	-2.3±0.1	0.98	70				
W High	3795.1±33.5	0.143±0.004	-1.75±0.07	0.99	92				
TRHA-/+ High	4067.1±43.4	0.108±0.003	-2.16±0.09	0.99	102				
TRHB-/+ High	4121.8±61.3	0.099±0.005	-2.3±0.2	0.992	70				
TRHC-/+ High	4144.2±71.3	0.09±0.004	-2.2±0.2	0.988	67				
TRHA-/- High	4096.4±86.9	0.092±0.005	-2.26±0.15	0.98	105				
TRHB-/- High	3953.2±98.3	0.092±0.006	-2.3±0.18	0.97	98				
TRHC-/- High	4057±132.4	0.086±0.008	-2.6±0.3	0.993	67				
TRHD-/- High	4332.9±151.6	0.073±0.006	-2.8±0.2	0.98	70				
**SSRI Study**									
W Low	3071.9±26.7	0.233±0.01	-1.3±0.07	0.99	34	I	1,68	2622.1	<0.001
TRH-/+ Low	2966.8±24.9	0.28±0.014	-1.03±0.07	0.99	34	C	3,68	25.1	<0.001
TRHA-/- Low	3219.1±105.6	0.151±0.018	-1.63±0.2	0.98	33	FX	1,68	0.8	0.366
TRHB-/- Low	3185.6±59.3	0.169±0.012	-1.51±0.13	0.99	36	IxC	3,68	85.7	<0.001
W Low+FX	3015.6±54.6	0.235±0.021	-1.33±0.14	0.98	32	IxFX	1,68	4.4	0.04
TRH-/+ Low+FX	3080.6±33	0.243±0.013	-1.14±0.09	0.99	35	CxFX	3,68	3.1	0.031
TRHA-/- Low+FX	3464.7±141.1	0.121±0.014	-1.87±0.2	0.99	34	IxCxFX	3,68	2.5	0.067
TRHB-/- Low+FX	3273.2±69.9	0.152±0.011	-1.63±0.14	0.99	35				

von Bertalanffy regression parameters (Mean ±SE) ,r^2 and sample size N and two factor repeated measure ANOVA testing for the effect of the repeated measure (adult instar, I), food ration (F) or fluoxetine (FX) and clone (C) on body length. All regressions curves and their parameters were significant p<0.001.

Table S5. Life-history results for seven studied clones in the food study showed reduced fecundity at high food levels, reduced offspring size and population growth rates, and delayed reproduction in bi-allelic indel mutated TRH clones lacking serotonin.

	N	MI (%)	N	Age (days)	N	Fec	N	Off Size (µm)	N	r (day-1)
W Low	20	25	20	9.1±0.1	19	21.9±0.5	19	889.2±4.5	20	0.259±0.004
TRHA-/+ Low	20	35	20	9.6±0.2	20	22.9±0.5	20	909±9.6	20	0.261±0.004
TRHB-/+ Low	10	100*	10	10.5±0.2*	10	23.3±0.7	10	912.4±11.7	10	0.248±0.004
TRHC-/+ Low	10	100*	10	10.5±0.2*	9	24.3±1.0*	10	881.8±7.3	10	0.251±0.005
TRHA-/- Low	20	100*	20	10.5±0.2*	20	23.2±0.4	18	857.5±9.2*	20	0.244±0.003*
TRHB-/- Low	20	100*	20	11±0.2*	20	23.7±0.5	18	842±9.8*	20	0.239±0.003*
TRHC-/- Low	10	100*	10	11.4±0.5*	9	23.1±0.5	9	867.1±6.7*	10	0.221±0.01*
TRHD-/- Low	10	100*	10	11.0±0.0*	10	24.1±0.6	10	829.4±11.3*	10	0.227±0.003*
W High	20	0	20	8.5±0.1	20	48.3±0.8	20	792.9±10.6	20	0.342±0.003
TRHA-/+ High	20	30*	20	9.3±0.2*	20	47.6±1.4	20	793.9±4.2	20	0.313±0.002*
TRHB-/+ High	10	0	10	9.5±0.0*	10	50.2±1.0	10	783.6±5.3	10	0.301±0.002*
TRHC-/+ High	10	100*	10	10.0±0.0*	10	51.3±3.0	10	762.9±4.4*	10	0.298±0.003*
TRHA-/- High	10	100*	10	9.7±0.2*	20	41.2±1.5*	20	763.5±4.9*	10	0.296±0.005*
TRHB-/- High	18	100*	18	10.5±0.3*	18	39.4±1.3*	17	774.3±4.9*	18	0.275±0.008*
TRHC-/- High	10	100*	10	12.4±0.3*	9	37.3±1.9*	9	757.4±12.7*	10	0.215±0.013*
TRHD-/- High	10	100*	10	11.4±0.3*	10	40.1±1.6*	8	758.4±9.9*	10	0.235±0.009*

This table included results from two replicated experiments (Experiment 1,2) which tested life-history performance of the studied clones at low and high food. Mean vales (±SE) for von Bertalanffy grow rate parameter (K), percentage of individuals investing more than five juvenile instars to maturity (MI, first releasing of eggs into the brood pouch), age at first reproduction, cumulative offspring production of the first three broods (Fec), offspring size (Off size), population growth rates (r) of *D. magna* individuals from the studied clones. Within a particular food environment * indicates significant ($p<0.05$) differences from the wild type clone following ANOVA and Dunnett's or the equivalent non parametric post-hoc comparison tests. Stats are depicted in Table S3

Supplementary Table S6. **Physiological responses of three of the studied clones in the food study. Stats results are also included.**

	N	Feeding	N	Oxygen	N	Lipid droplets
W	16	100.1±1.6	10	100±4.1	19	100±8.1
TRHA+/-	14	93.8±3.1	9	92.6±4.1	21	101.4±9.3
TRHA-/-	16	99.8±2.2	10	100±1.3	14	96.3±7.4
TRHB-/-	13	93.6±2.1	10	105.2±1.8	16	94±8

Mean (±SE) proportional responses relative to the wild type clone of feeding rates, oxygen consumption rates and lipid droplets of the studied clones cultured at the high food ratio. N, sample size. Results from two independent experiments were pooled.

Supplementary Table S7. **Statistical results for the SSRI study**.

	Factor	df	Test	p
Maturation	C ($\chi 2$)	3	28.3	<0.001
	FX($\chi 2$)	1	0.3	0.795
Age	C (KW)	7	46.6	<0.001
	FX(MW)		705.5	0.857
	Factor	df	F	
Swimming	Ph	1,314	41	<0.001
	FX	1,314	0.4	0.528
	C	3,314	53.6	<0.001
	Ph x FX	1,314	6.3	0.013
	Ph x C	3,314	4.8	0.003
	FX x C	3,314	4.4	0.004
	Ph x FX x C	3,314	1.1	0.355
Fecundity	C	3,67	9.2	<0.001
	FX	1,67	47.3	<0.001
	CxFX	3,67	0.7	0.536
Offsring size	C	3,64	39.7	<0.001
	FX	1,64	1.3	0.258
	CxFX	3,64	5.5	0.002
r	C	3,67	29.6	<0.001
	FX	1,67	0.6	0.45
	CxFX	3,67	0.8	0.518

Chi square ($\chi 2$) , Kruskal-Wallis(KW) and Mann-Whitney (MW)test results testing for effects of clone (C) and fluoxetine (FX) exposure on the % of individuals investing more than 5 juvenile instars to maturity (Maturation) and age at first reproduction, respectively. ANOVA results testing for: the effects of fluoxetine (FX) and clone on total offspring production (fecundity), offspring size and population growth rates (r); for effects of photoperiod (Ph), fluoxetine and clone on swimming distance (swimming). df, F, p are degrees of freedom, Fisher's coefficient and probability level, respectively.

Supplementary Figures

5’- GCGAAACGAGTGAACGTCGTTCACATTGAATCACGCCC**GTCAATGCGTCGCAATTC**CCAGTAC
GAGATCATGGTGGATGTCCAATGCAACGATGATCAAATGACCGACCTCATCGCTTCGCTTCAAAATGAAGTA
GCTGCTGTTAAACTGGCCGAATTCGACATGGGACTCGATCCTCCAATGTCACCGGCCATCAGTGAAAGCTTC
GAAAATTTTGAGGACATGATCTGGTTCCCGAGACGCATCTCCGAATTGGACCAGGCTCAGCGGGTTTTGCTT
TACGGTGCGG**ATTTAGACGCCGACCATC**CGGTGAGTGCAACCCAATCACGCATCTATAAGCCATGCAATAAG
ACTAATAATCTATGATTGGCTTTCAAAAAGGGATTCAAAGACCCCGTGTACCGTAAAAGGCGAAAGTACTTC
ACAGACTTGGCCATGGCTTATCGCTAGTAAGTGAATATCCATTTTTATGTTATGGGTTTTTATCTTTATGTGCT
GAGACCTAATCAAATCTTACGTCACTTTACTTGCTGCGTACTTTTTGATCCGACTACTTGATTGAATTTCTGCG
ATAAAAGTGGAGAGCCTATTCCGAGAGT-3’

Supplementary Figure S1. R**esults of PCR for cloned Partial cDNA sequence of D. magna *DapmaTRH*** . Cutting sites are depicted in red.

Studied clones
Wild type
GCGAAACGAGTGAACGTCGTTCACATTGAATCACGCCCGTCAATGCGTCGCAAT
TCCCAGTACGAGATCATGGTGGATGTCCAATGCAACGATGATCAAATGACCGAC
CTCATCGCTTCGCTTCAAAATGAAGTAGCTGCTGTTAAACTGGCCGAATTCGAC
ATGGGACTCGATCCTCCAATGTCACCGGCCATCAGTGAAAGCTTCGGTAAATTA
TAGTTGCTCCTCTAGCAACATTCACTTTGTCCGATTTAGGACTAACTGTTGAATC
TTCTTGTAAAAGAAAATTTTGAGGACATGATCTGGTTCCCGAGACGCATCTCCG
AATTGGACCAGGCTCAGCGGGTTTTGCTTTACGGTGCGGATTTAGACGCCGACC
ATCCGGTGAGTGCAACCCAATCACGCATCTATAAGCCATGCAATAAGACTAATA
ATCTATGATTGGCTTTCAAAAAGGGATTCAAAGACCCCGTGTA

TRHA-/+
allele 1:(-4,+0)
GCGAAACGAGTGAACGTCGTTCACATTGAATCACGCCCGTCAATGCGTCGCAAT
TCCCAGTACGAGATCATGGTGGATGTCCAATGCAACGATGATCAAATGACCGAC
CTCATCGCTTCGCTTCAAAATGAAGTAGCTGCTGTTAAACTGGCCGAATTCGAC
ATGGGACTCGATCCTCCAATGTCACCGGCCATCAGTGAAAGCTTCGGTAAATTA
TAGTTGCTCCTCTAGCAACATTCACTTTGTCCGATTTAGGACTAACTGTTGAATT
TTCTTGTAATAGAAAATTTTGAGGACATGATCTGGTTCCCGAGACGCATCTCCG
AATTGGACCAGGCTCAGCGGGTTTTGCTTTACGGTGCGGATTTAGACGCCGACC
GGTGAGTGCAACCAAATCACGCATCTATAAGCCATGCAATAAGACTAATAATCT
ATGATGGCTTTCAAAAAGGGATTCAAAGACCCCGTGTA

TRHB-/+
allele1:(-7,+0)
GCGAAACGAGTGAACGTCGTTCACATTGAATCCGCCCGTCAATGCGTCGCAATT
CCCAGTACGAGATCATGGTGGATGTCCAATGCAACGATGATCAAATGACCGACC
TCATCGCTTCGCTTCAAAATGAAGTAGCTGCTGTTAAACTGGCCGAATTCGACA
TGGGACTCGATCCTCCAATGTCACCGGCCATCAGTGAAAGCTTCGGTAAATTAT
AGTTGCTCCTCTAGCAACATTTACTTTGTCCGATTCAGGACTAACTGTTGAATCT
TCTTGTAAAAGAAAATTTTGAGGACATGATCTGGTTCCCGAGACGCATCTCCGA
ATTGGACCAGGCTCAGCGGGTTTTGCTTTACGGTGCGGATTTAGACGCCGAGTG
AGTGCAACCCAATCACGCATCTATAAGCCATGCAATAAGACTAATAATCTATGA
TTGGCTTTCAAAAAGGGATTCAAAGACCCCGTGT
TRHC-/+
allele1:(-3,+0)
GCGAAACGAGTGAACGTCGTTCACATTGAATCACGCCCGTCAATGCGTCGCAAT
TCCCAGTACGAGATCATGGTGGATGTCCAATGCAACGATGATCAAATGACCGAC
CTCATCGCTTCGCTTCAAAATGAAGTAGCTGCTGTTAAACTGGCCGAATTCGAC
ATGGGACTCGATCCTCCAATGTCACCGGCCATCAGTGAAAGCTTCGGTAAATTA
TAGTTGCTCCTCTAGCAACATTCACTTTGTCCGATTTAGGACTAACTGTTGAATT
TTCTTGTAATAGAAAATTTTGAGGACATGATCTGGTTCCCGAGACGCATCTCCG
AATTGGACCAGGCTCAGCGGGTTCTGCTTTACGGTGCGGATTTAGACGCCGACC
CGGTGAGGGCAACCAAATCACGCATCTATAAGCCATGCAATAAGACTAATAATC
TATGATGGCTTTCAAAAAGGGATTCAAAGACCCCGTGT

TRHA-/-
allele1:(-0,+5)
GCGAAACGAGTGAACGTCGTTCACATTGAATCACGCCCGTCGTGGGAATGCGTC
GCAATTCCCAGTACGAGATCATGGTGGATGTCCAATGCAACGACGATCAAATGA
TCGACCTCATCGCTTCGCTTCAAAATGAAGTAGCTGCTGTTAAACTGGCCGAATT
CGACATGGGACTCGATCCTCCAATGCCACCGGCCATCAGTGAAAGCTTCGGTAA
ATTATAGTTGCTCCTCTAGCAACATTCACTTTGTCCGATTTAGGACTAACTGTTG
AATTTTCTTGTAATAGAAAATTTTGAGGACATGATCTGGTTCCCGAGACGCATCT
CCGAATTGGACCAGGCTCAGCGGGTTTTGCTTTACGGTGCGGATTTAGACGCCG
ACCATCCGGTGAGTGCAACCAAATCACGCATCTATAAGCCATGCGATAAGACTA
ATAATCTATGATTGGCTTTCAAAAAGGGATTCAAAGACCCCGTGT
allele2:(-2,+15)
GCGAAACGAGTGAACGTCGTTCACATTGAATCACGCCCGTCGTTTCCTCCAAAT
GCGTCGCAATTCCCAGTACGAGATCATGGTGGATGTCCAATGCAACGATGATCA
AATGACCGACCTCATCGCTTCGCTTCAAAATGAAGTAGCTGCTGTTAAACTGGC
CGAATTCGACATGGGACTCGATCCTCCAATGTCACCGGCCATCAGTGAAAGCTT
CGGTAAATTATAGTTGCTCCTCTAGCAACATTTACTTTGTCCGATTCAGGACTAA
CTGTTGAATCTTCTTGTAAAAGAAAATTTTGAGGACATGATCTGGTTCCCGAGG
CGCATCTCCGAATTGGACCAGGCTCAGCGGGTTTTGCTTTACGGTGCGGATTTA
GACGCCGACGGATGTCCGGCGAGTGCAACCCAATCACGCATCTATAAGCCATGC
AATAAGACTAATAATCTATGATTGGCTTTCAAAAAGGGATTCAAAGACCCCGTG
T

TRHB-/-
allele1:(-8,+6)
GCGAAACGAGTGAACGTCGTTCACATTGAATCACGCCCGTCAATGCGTCGCAAT
TCCCAGTACGAGATCATGGTGGATGTCCAATGCAACGATGATCAAATGACCGAC
CTCATCGCTTCGCTTCAAAATGAAGTAGCTGCTGTTAAACTGGCCGAATTCGAC
ATGGGACTCGATCCTCCAATGTCACCGGCCATCAGTGAAAGCTTCGGTAAATTA
TAGTTGCTCCTCTAGCAACATTCACTTTGTCCGATTTAGGACTAACTGTTGAATT
TTCTTGTAATAGAAAATTTTGAGGACATGATCTGGTTCCCGAGACGCATCTCCG
AATTGGACCAGGCTCAGCGGGTTTTGCTTTACGGTGCGGATTTAGACGCCGACG
CCGACGAGTGCAACCAAATCACGCATCTATAAGCCATGCAATAAGACTAATAAT
CTATGATGGCTCTCAAAAAGGGATTCGAAGACCCCGTGTAA

allele2:(-8,+0)
GCGAAACGAGTGAACGTCGTTCACATTGAATCACGCCCGTCAATGCGTCGCAAT
TCCCAGTACGAGATCATGGTGGATGTCCAATGCAACGACGATCAAATGACCGAC
CTCATCGCTTCGCTTCAAAATGAAGTAGCTGCTGTTAAACTGGCCGAATTCGAC
ATGGGACTCGATCCTCCAATGTCACCGGCCATCAGTGAAAGCTTCGGTAAATTA
TAGTTGCTCCTCTAGCAACATTTACTTTGTCCGATTCAGGACTAACTGTTGAATC
TTCTTGTAAAAGAAAATTTTGAGGACATGATCTGGTTCCCGAGACGCATCTCCG
AATTGGACCAGGCTCAGCGGGTTTTGCTTTACGGTGCGGATTTAGACGCCGGTG
AGTGCAACCCAATCACGCATCTATAAGCCATGCAATAAGACTAATAATCTATGA
TTGGCTTTCAAAAAGGGATTCAAAGACCCCGTGT

TRHC-/-
allele1:(-8,+0)
GCGAAACGAGTGAACGTCGTTCACATTGAATCACGCCCGTCGCAATTCCCAGTA
CGAGATCATGGTGGATGTCCAATGCAACGATGATCAAATGACCGACCTCATCGC
TTCGCTTCAAAATGAAGTAGCTGCTGTTAAACTGGCCGAATTCGACATGGGACT
CGATCCTCCAATGTCACCGGCCATCAGTGAAAGCTTCGGTAAATTATAGTTGCT
CCTCTAGCAACATTTACTTTGTCCGATTCAGGACTAACTGTTGAATCTTCTTGTA
AAAGGAAAATTTTGAGGACATGATCTGGTTCCCGAGACGCATCTCCGAATTGGA
CCAGGCTCAGCGGGTTTTACTTTACGGTGCGGATTTAGACGCCGACCATCCGGT
GAGTGCAACCCAATCACGCATCTATAAGCCATGCAATAAGACTAATAATCTATG
ATTGGCTTTCAAAAAGGGATTCAAAGACCCCGTGTA
allele2:(-2,+27)
GCGAAACGAGTGAACGTCGTTCACATTGAATCCGCCCGTCAATGCGTCGCAATT
CCCAGTACGAGATCATGGTGGATGTCCAATGCAACGATGATCAAATGACCGACC
TCATCGCTTCGCTTCAAAATGAAGTAGCTGCTGTTAAACTGGCCGAATTCGACA
TGGGACTCGATCCTCCAATGTCACCGGCCATCAGTGAAAGCTTCGGTAAATTAT
AGTTGCTCCTCTAGCAACATTCACTTTGTCCGATTTAGGACTAACTGTTGAATTT
TCTTGTAATAGAAAATTTTGAGGACATGATCTGGTTCCCGAGACGCATCTCCGA
ATTGGACCAGGCTCAGCGGGTTTTGCTTTACGGTGCGGATTTAGACGCCGCGAG
TGCAACCAAAGACGCACCGAGTGCATCCGGTGAGTGCAACCAAATCACGCATCT
ATAAGCCATGCAATAAGACTAATAATCTATGATTGGCTTTCAAAAAGGGATTCA
AAGACCCCGTGTA
TRHD-/-
allele1:(-10,+2)
GCGAAACGAGTGAACGTCGTTCACATTGAATCACGCCCGTCGTAATTCCCAGTA
CGAGATCATGGTGGATGTCCAATGCAACGATGATCAAATGACCGACCTCATCGC
TTCGCTTCAAAATGAAGTAGCTGCTGTTAAACTGGCCGAATTCGACATGGGACT
CGATCCTCCAATGTCACCGGCCATCAGTGAAAGCTTCGGTAAATTATAGTTGCT
CCTCTAGCAACATTCACTTTGTCCGATTTAGGACTAACTGTTGAATTTTCTTGTA
ATAGAAAATTTTGAGGACATGATCTGGTTCCCGAGACGCATCTCCGAATTGGAC
CAGGCTCAGCGGGTTTTGCTTTACGGTGCGGATTTAGACGCCGACCATCCGGTG
AGTGCAACCAAATCACGCATCTATAAGCCATGCAATAAGACTAATAATCTATGA
TGGCTTTCAAAAAGGGATTCAAAGACCCCGTGT
allele 2: due to a putative large deletion and/or the formation of secondary DNA structures (Watanabe, personal communication) it is not always possible to sequence this allele.

Supplementary Figure S2. Genomic *DapmaTRH* sequences of the wild type clone and the seven ones **obtained by CRISPR-Cas9.** The length (in base pairs) of each indel mutation is marked on the left of each sequence (- stands for deletions; + for insertions)

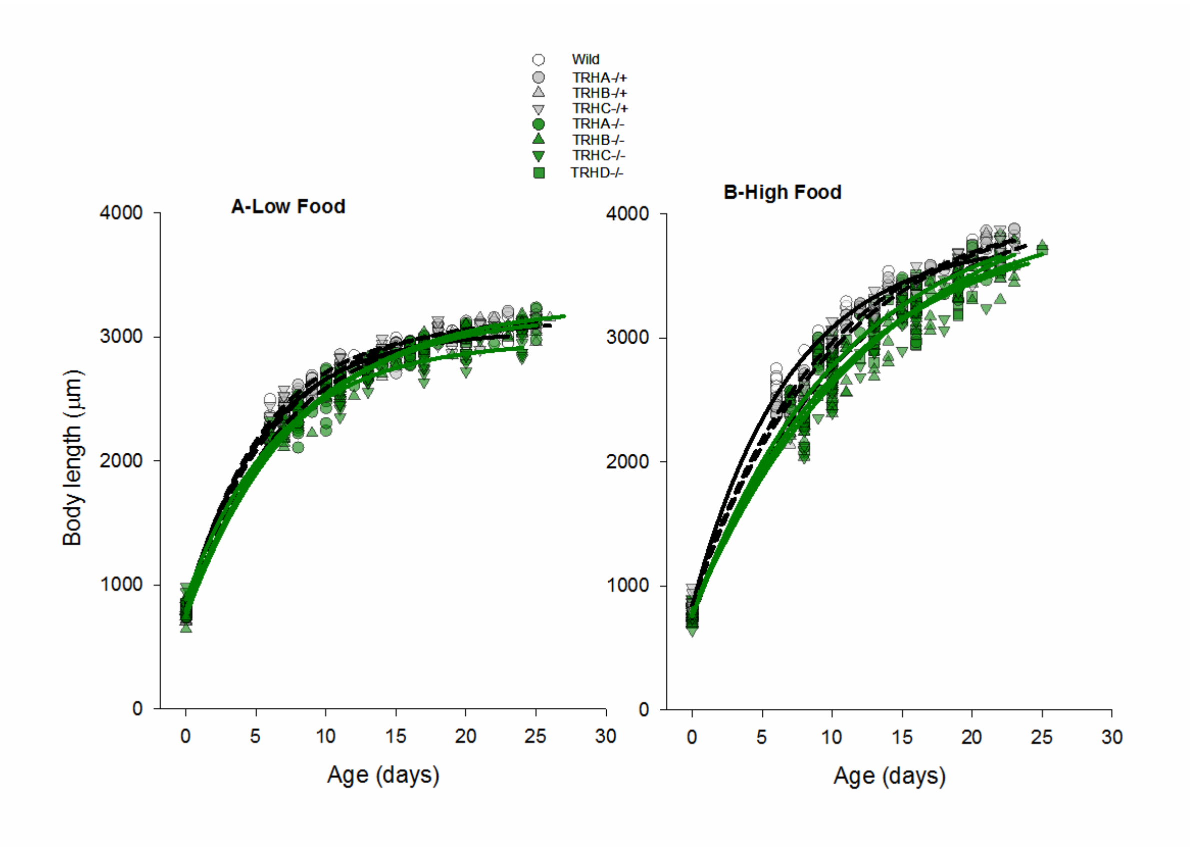

Figure S3. Bi-allelic indel mutated TRH clones lacking serotonin grew less than the mono-allelic mutated TRH and wild ones. Body length measurements and fitted von Bertalanffy growth for *D. magna* individuals of the studied clones cultures under low (A) and high (B) food ratios. Continuous and dashed black lines correspond to fitted curves for wild and TRH-/+ clones, respectively, whereas green lines to TRH-/- ones.

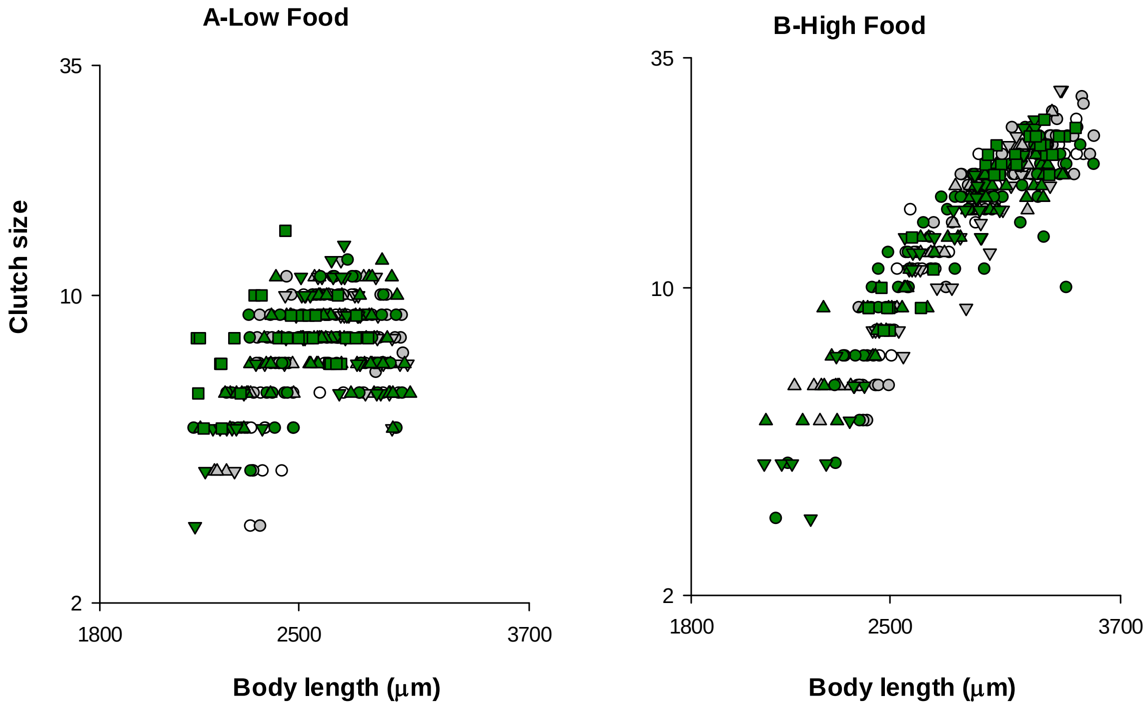

Supplementary Figure S5. **Differences in body length among females from the studied clones accounted for the observed differences in fecundity.** Clutch size versus body length of *D. magna* individuals from the studied clones at Low (A) and High food levels (B). Data from the two experiments were pooled. Each symbol corresponds to a single individual value. Axis are plotted in log scale. Results from ANCOVA for the covariate body length (BL) and clone (C) were:

BL, $F_{1,350}$ =28.4, <0.001; C, $F_{7,350}$ =1.6, p =0.1, Low Food

BL, $F_{1,344}$ =1971.3, p <0.001; C, $F_{7,344}$ =0.9, p=0.5, High Food

Supplementary Methods

CRISPR-Cas9 mediated targeted mutagenesis

There is only one probable orthologue of mammalian tryptophan hydrolase in the D. magna genome (Dapma7bEVm006764t1 hereafter referred as TRH, scaffold00084:361112-363778), which contains 15 exons (Fig 1). Reverse transcription PCR of the TRH gene using a primer set encompassing the targeted mutagenic sites (Table S1) as well as sequencing of the PCR fragments revealed that this gene is transcribed in *D. magna* (Figure S1). Then CRISPR-Cas9 targeted mutagenesis was performed according to Nakanishi, et al. [1]. Briefly, for the synthesis of Cas 9 mRNAs, templates with T7 promoter were amplified by PCR using PrimeSTAR (Takara Bio, Shiga, Japan) from the pCS-Dmavas-Cas9. Amplified PCR fragments were subjected to in vitro transcription with the mMessage mMachine T7 kit (Life Technologies, California, USA). Poly (A) tails were attached to capped Cas9 RNAs by using a Poly(A) Tailing Kit (Life Technologies, California, USA) and purified. For the synthesis of gRNAs, four oligonucleotides (two for each TRH target site encompassing exons 3 and 4, respectively, Table S2) were annealed and then ligated into the linearized pDR274 vector (Addgene plasmid 42250) using a ligation mix (TaKaRa Bio, Shiga, Japan). Oligonucleotides for knocking down the TRH gene were designed using ZiFiT targeter version 4.2 [2]. The two genomic targeted mutagenesis sites and sequences of the oligonucleotides used in this study are listed in Figure 1. pDR274-TRH vectors were then digested by DraI and used as templates for in vitro transcription with the mMessage mMachine T7 kit, followed purification. By using a BLASTn search on the *D. magna* genome database (wfleabase.org), we looked for potential off-target sites and found that TRH-1 and TRH-2 target sites had no potential off-target sites.

Cas9 and gRNAs were co-injected into Daphnia eggs as performed in Kato, et al. [3]. Briefly, eggs were collected immediately after ovulation from 2 to 3 weeks old daphnids and kept in

ice-chilled M4-sucrose 80 mM. Injection was performed through a glass needle under N_2 gas pressure. The injection volume was approximately 0.2 nL and microinjections were carried out within an hour after ovulation. Injected eggs were then kept in M4-sucrose for 60 hours and embryonal development was monitored. After first and second generation PCR amplification of target loci were performed on genomic DNA extracted from alive mutant clonal lines to characterize Cas9-induced mutations. Genomic DNA was extracted from single daphniids by homogenization in 90 mL of 50 mM NaOH. The lysate was heated at 95 ^{o}C for 10 min and then neutralized with 10 mL of 1 M Tris-HCl (pH 7.5). This crude DNA extract was centrifuged at 10,000 g for 5 min prior to being used as a template for genomic PCR. All PCRs were performed with ExTaq DNA polymerase, Hot Start version (Takara). The PCR products were analyzed firstly by polyacrylamide gel electrophoresis (PAGE) and selected lines were DNA sequenced using the Sanger method. The primers used for PCR and DNA sequencing are also listed in Table S4 .

Feeding

Feeding assays were conducted with groups of five juveniles transferred into individual test vessels filled with 100 mL of media at high food ration level (5 x 10^5 cells/ml C. vulgaris). Two replicate vessels filled with the same culture medium but with no animals were used as blank replicates. The mean initial cell concentration of the experimental vessels at the start of the experiment (time t0) was determined from three 5-ml samples obtained from the treatment medium before it was distributed into all the experimental vessels. After 24 h, the final cell concentration (time t24) was measured. Feeding was then measured using the cell-difference method following Barata and Baird [4] and expressed as proportional responses relative to controls.

Oxygen determination

Oxygen consumption assays were performed without food using standard respirometry methods with 50 ml gas-tight syringes (Hamilton, USA) as described by Agra, et al. [5], and expressed as expressed as proportional responses relative to the wild type clone.

Storage lipid accumulation

Quantification of storage lipids into lipid droplets follow previous methods [6]. Nile red stock solutions were prepared in acetone and store protected from light. Just before use, the working solution was obtained by dilution of stock solution to 1.5 µM in ASTM. Live individuals were then exposed to Nile red working solution in the dark for 1 h at 20 ºC. After incubation, animals were place in 100 ml ASTM for 1 min to allow clearance of Nile red residuals. Following clearance animals were placed individually in 1.5 ml centrifuge tubes, the remaining water removed and sonicated in 300 µl of isopropanol. The homogenized extract was then centrifuged at 10 000 g. We used 200 µl of supernatant to measure Nile red fluorescence using an excitation/emission wavelength 530/590 nm and a microplate fluorescence reader (Synergy 2, BioTek, USA). For each quantification and treatment 10 blanks (non-exposed animals to Nile red) were used to account for background levels of fluorescence. Nile red fluorescence was expressed as proportional responses relative to the wild type clone.

Brain whole-mount immunofluorescence

Methods for immunofluorescence microscopy were performed as described previously [7,8]. In brief brains were dissected in 0.1M phosphate buffered saline (PBS) and immersion-fixed in 4 % paraformaldehyde in PBS overnight at room temperature. The fixed brains were first washed with PBS three times for five minutes. To quench remaining fixative, brains were then washed and permeabilized with 0.1M Tris-hydroxylamino-methane buffered saline containing 0.5 % Triton X100 (TBTX) four times for 10 minutes. Samples were then incubated overnight at room temperature with the primary antibody rabbit anti-serotonin

(Immunostar 20080) diluted 1:12,000 in TBTX with 0.02 % sodium azide. After washing with TBTX, the samples were incubated for 1 h with a secondary goat-anti-rabbit-FITC-conjugated antibody (Sigma F0382) diluted 1:100 in TBTX. After three washes of 10 minutes with TBTX, the brains were mounted on glass slides in 80 % aqueous glycerol containing 50 mg/mL diaza-bicyclo-octane (DABCO) (Fluka) as anti-fade. Fluorescence images of D. magna brains were obtained using a Nikon Eclipse 90i microscope fitted with a Nikon Intensilight C-HGFI unit. Images were acquired with a Nikon Digital Sight DS-Ri1 camera and NIS Elements AR software (version 3.0) and saved as high-resolution (3840 pixels x 3005 pixels) tagged image file format (TIFF). All image series of every experiment were taken the same day, using the same settings.

Neurotransmitter analysis

Pure analytical standards of 99% purity of Methanol (MeOH), acetonitrile (ACN), and HPLC water (LiChrosolv grade) were supplied by Merck (Darmstadt, Germany). All of the analytical standards of acetylcholine, dopamine, epinephrine, γ- aminobutyric acid (GABA), norepinephrine, octopamine and serotonin were purchased from Sigma-Aldrich (98-99% purity, Schnelldorf, Germany). The stable isotope-labeled internal standards of acetylcholine-d9, GABA-d6 and serotonin-d4 were bought from Toronto Research Chemicals (Toronto, Ontario, Canada), whereas dopamine-d4 was from Sigma-Aldrich (Schnelldorf, Germany). Stock solutions for all compounds were prepared in Milli-Q water with 0.1% of formic acid at a concentration of 1000 ppm. Final working dilutions were made in ACN : H2O (1:1, v/v) with 0.1% of formic acid. In the preparation of the standards, an exhaustive control on handling procedures, storage conditions and safety rules has been followed, as specified by manufacturers. Four replicates of five 4-days-old daphnids were collected , snap-frozen dry and stored at −80 °C until analysis. Samples were homogenized in ice-cold ACN with 0.1% of formic acid with the addition of neurotransmitter stable

isotope-labeled internal standards. Samples were then centrifuged to remove animal debris, before protein precipitation was allowed at -20C for 30 min. Finally, samples were centrifuged at 14.500 rpm for 10 min, evaporated to dryness under N2 current and resuspended in 50 µL ACN : H2O (1:1, v/v). A further step of 10 min centrifugation at 10 000 g was included to ensure sample purity before injection. All steps were carried out at 4C and in dark conditions. Analysis of neurotransmitters was performed by liquid chromatography-tandem mass spectrometry following Tufi, et al. [9] with some minor modifications. Briefly, analyte separation was obtained by using a TSK GelAmide 80 HILIC column (2 × 250 mm, 5 µm particle size, Sigma Aldrich) and the analyses was carried out using a TqDetector (Acquity Waters, USA). The mobile phase composition consisted of binary mixtures with ACN : H_2O (90:10, v/v) (A) and water (B), both buffered with 10 mM of ammonium formate and 0,1% formic acid. The gradient of elution started at 0% B , then increased to 30% B in 8 min and kept for another 2 min, then increased to 45% B in 4 min and kept for 3 min, before returning to initial conditions in 2 min for re-equilibration. Total run time was set to 25 min. The system was operated at room temperature, the flow rate was set at 150 µL min−1 and 10 µL were injected. Acquisition was performed in SRM mode under positive electrospray ionization (ESI+) using two transitions from [M+H]+ precursor ion to daughter ions. The transitions used as well as the cone voltages and collision energies used for each analyte are presented in Table S8. Data was acquired and processed using the MassLynx v4.1 software package. Quantification was based on the most intense transition of each analyte. Stable isotope-labeled internal standards were used for calibration and quantification. For the method validation several parameters were determined and are depicted in Table S9: inter- and intra-day variation, linearity, limit of detection (LOD) and limit of quantification (LOQ). LOD and LOQ were defined as the minimum detectable amount of analyte with a signal to noise ratio (RMS) of 3:1 and 10:1, respectively.

Table S8. MRM transitions and MS/MS parameters of the compounds and stable isotope-labelled internal standards.

Compound	Labeled IS	Parent ion (m/z)	Quantifier (m/z)	Qualifier (m/z)	Cone voltage (V)
Acetylcholine	Acethylcholine-d9	146.3	87.1 (13)	60.1 (11)	18
Acethylcholine-d9		155.3	87.1 (16)	43.1 (26)	28
Dopamine	Dopamine-d4	154.2	137.2 (13)	119 (22)	20
Dopamine-d4		158.3	141.3 (15)	95.2 (25)	30
Epinephrine	Dopamine-d4	184.3	166.1 (10)	107 (21)	15
GABA	GABA-d6	104.2	87.1 (8)	69.1 (14)	15
GABA-d6		110.2	93.1 (11)	49.1 (17)	35
Norepinephrine	Dopamine-d4	152.2	135.1 (14)	107.1 (15)	28
Octopamine	Dopamine-d4	154.3	136.1 (9)	91 (25)	14
Serotonin	Serotonin-d4	177.2	160.2 (10)	132.1 (21)	16
Serotonin-d4		181.3	164.1 (11)	136 (21)	36

Collision energies (eV) for quantifier, qualifier ions are depicted in brackets

Table S9. Validation parameters of the analytical method

Analyte	RT (min)	RSD (%) Intraday	RSD (%) interday	Linearity ng/mL	R^2	Recovery (%) Mean ± SD	LOD ng/mL	LOQ ng/mL
Acetylcholine	8.54	3.6	8.6	0.09-900	0.99	81.4+5.9	0.02	0.05
Dopamine	8.79	7.3	11.4	0.45-900	0.99	72.2+20.5	0.48	1.6
Epinephrine	9.15	7.2	9.7	0.45-900	0.99	73+14.6	0.1	0.34
Norepinephrine	9.58	3.1	19.5	0.9-900	0.99	76+8.5	0.3	0.99
Octopamine	8.79	2.9	4.3	0.45-900	0.99	78+14.6	0.33	1.09
GABA	10.52	1.3	11.0	4.5-900	0.99	112.9+21.1	1.71	5.69
Serotonin	8.16	6.2	7.1	0.45-900	0.99	70.6+3.1	0.03	0.07

References

1 Nakanishi, T., Kato, Y., Matsuura, T. & Watanabe, H. CRISPR/Cas-mediated targeted mutagenesis in Daphnia magna. *PLoS ONE* **9**, doi:10.1371/journal.pone.0098363 (2014).

2 Sander, J. D. *et al.* ZiFiT (Zinc Finger Targeter): An updated zinc finger engineering tool. *Nucleic Acids Research* **38**, doi:10.1093/nar/gkq319 (2010).

3 Kato, Y. *et al.* Development of an RNA interference method in the cladoceran crustacean Daphnia magna. *Development Genes and Evolution* **220**, 337-345, doi:10.1007/s00427-011-0353-9 (2011).

4 Barata, C. & Baird, D. J. Determining the ecotoxicological mode of action of toxicants from measurements on individuals: results from short duration chronic tests with *Daphnia magna* Straus. *Aquatic toxicology* **48**, 195-209 (2000).

5 Agra, A. R., Soares, A. & Barata, C. Life-history consequences of adaptation to pollution. "Daphnia longispina clones historically exposed to copper". *Ecotoxicology* **20**, 552-562, doi:10.1007/s10646-011-0621-5 (2011).

6 Jordão, R. *et al.* Obesogens beyond vertebrates: Lipid perturbation by tributyltin in the crustacean Daphnia magna. *Environmental Health Perspectives* **123**, 813-819, doi:10.1289/ehp.1409163 (2015).

7 Dircksen, H., Tesfai, L. K., Albus, C. & Nässel, D. R. Ion transport peptide splice forms in central and peripheral neurons throughout postembryogenesis of *Drosophila melanogaster*. *The Journal of comparative neurology* **509**, 23-41, doi:10.1002/cne.21715 (2008).

8 Campos, B., Rivetti, C., Kress, T., Barata, C. & Dircksen, H. Depressing Antidepressant: Fluoxetine Affects Serotonin Neurons Causing Adverse Reproductive Responses in Daphnia magna. *Environmental Science and Technology* **50**, 6000-6007, doi:10.1021/acs.est.6b00826 (2016).

9 Tufi, S., Lamoree, M., de Boer, J. & Leonards, P. Simultaneous analysis of multiple neurotransmitters by hydrophilic interaction liquid chromatography coupled to tandem mass spectrometry. *Journal of Chromatography A* **1395**, 79-87, doi:10.1016/j.chroma.2015.03.056 (2015).
